# Pilomatrixoma of the Eyelid in an 83-Year-Old Woman: A Rare Case in the Elderly

**DOI:** 10.7759/cureus.86187

**Published:** 2025-06-17

**Authors:** Dina Yazidi, Nicolas De Saint-Aubain, Sebastien Michiels

**Affiliations:** 1 Department of General Surgery, Centre Hospitalier Interrégional Edith Cavell (CHIREC), Brussels, BEL; 2 Department of Pathology, Jules Bordet Institut, Brussels, BEL

**Keywords:** benign skin neoplasm, eyelid tumor, geriatric oncology, head and neck tumor, pilomatrixoma

## Abstract

Pilomatrixoma is a rare subcutaneous tumor originating from the cellular matrix of hair follicles. While it predominantly arises during the first two decades of life, its occurrence in elderly patients is exceedingly rare. We present the case of an 83-year-old woman with a recently growing, indurated nodule located on the right upper eyelid. Her medical history included left breast cancer treated with tumorectomy, radiotherapy, and chemotherapy, with remission since 2012. Given her advanced age and oncological history, differential diagnoses included malignant tumors, metastasis, or recurrence of breast cancer involving the head and neck region. Histopathological analysis confirmed the diagnosis of pilomatrixoma, which was completely excised with clear margins. The postoperative course was uneventful, and no recurrence was observed at one-month follow-up. This case highlights the importance of including pilomatrixoma in the differential diagnosis of head and neck lesions in elderly patients.

## Introduction

Pilomatrixoma is a benign subcutaneous tumor derived from the hair follicle matrix and primarily affects children and adolescents, with a marked female predominance [1,2]. Lesions typically occur in the head and neck area but may also arise, less frequently, in the upper extremities, trunk, and lower extremities [1,2]. It belongs to the group of benign skin adnexal tumors, which include other lesions derived from sweat glands, sebaceous glands, or hair follicles. Although clinical diagnosis is sometimes possible, histological confirmation remains the gold standard. Surgical excision is curative, and recurrence is rare [3,4]. Although pilomatrixoma can occur at any age, its appearance in the elderly remains exceptionally rare and may present diagnostic challenges due to the broader differential in this age group. Furthermore, in elderly patients, the potential for malignant transformation or confusion with metastatic lesions, especially in oncologic contexts, warrants careful evaluation. In this report, we present a rare case of pilomatrixoma in an elderly patient, highlighting its clinical presentation, histological features, and differential diagnosis considerations.

## Case presentation

An 83-year-old woman was referred to the general surgery department for evaluation of a painless, firm, and mobile subcutaneous nodule in the right eyebrow. The lesion was located in the lateral portion of the right upper eyelid, just superior to the tarsal plate. It had remained stable in size for several years before gradually enlarging over the past two months. Her medical history included left breast carcinoma treated with tumorectomy, axillary lymphadenectomy, and adjuvant chemo-radiotherapy in 2012, with no recurrence since.

On physical examination, the nodule was firm, non-tender, and mobile relative to deeper planes, measuring 1 cm × 1.5 cm with well-defined margins. No additional cutaneous abnormalities, no regional lymphadenopathy, or systemic signs of malignancy were detected.

**Figure 1:**
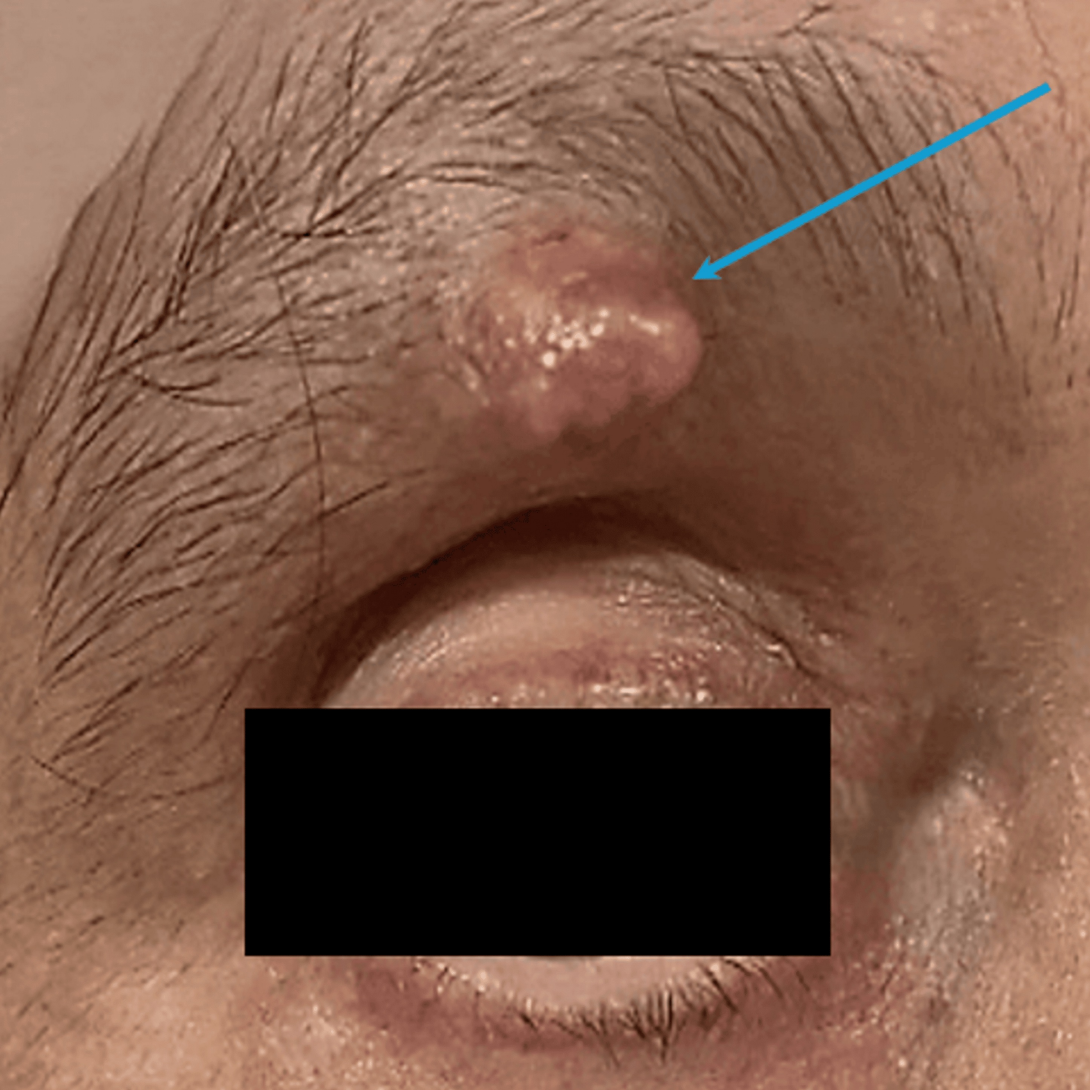
Clinical appearance of the eyelid lesion prior to excision (blue arrow).

**Figure 2:**
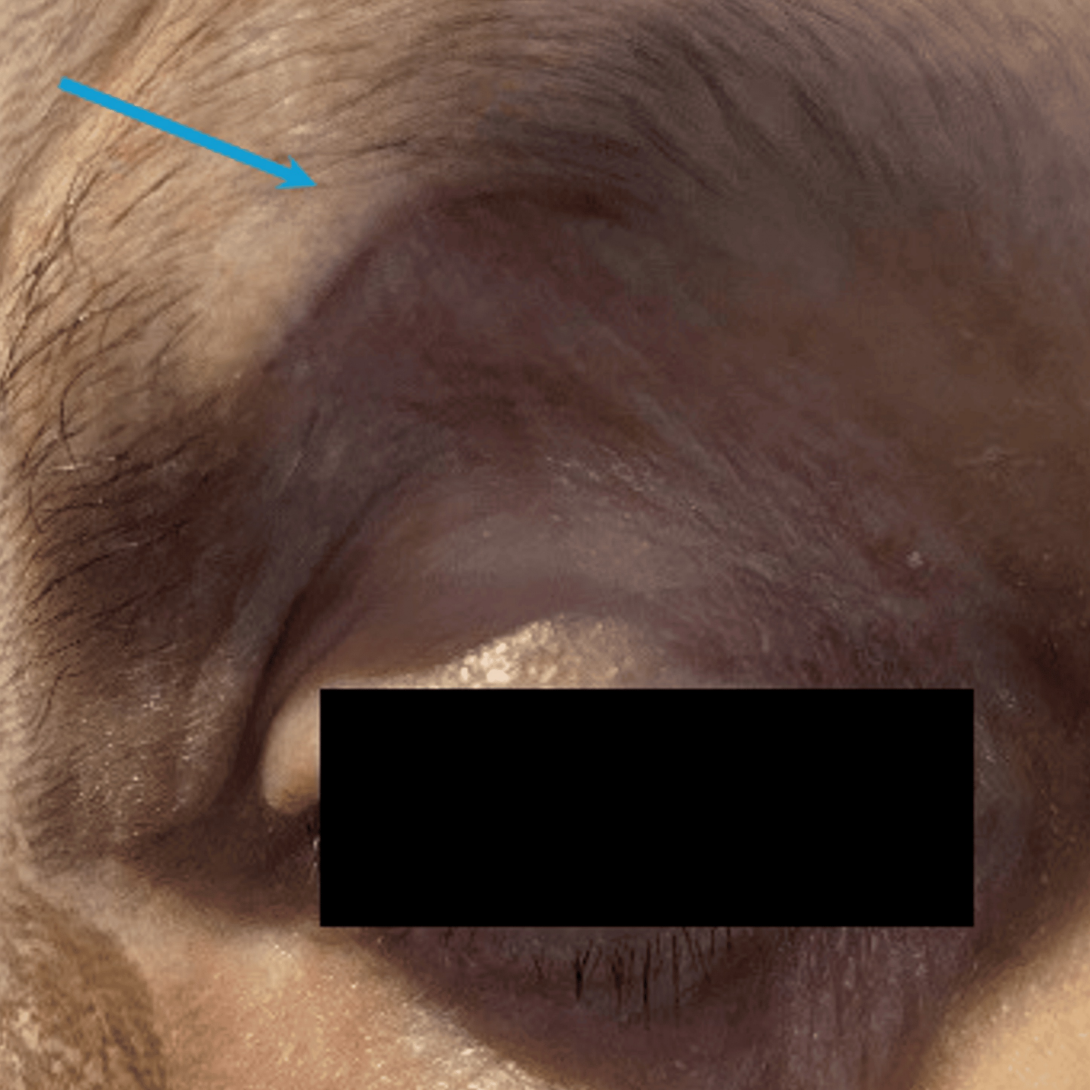
Surgical site two weeks postoperatively (blue arrow)

In light of the clinical impression of a benign lesion, no additional imaging or fine-needle aspiration was performed. Bilateral breast examination was unremarkable, with no masses, lymphadenopathy, nipple discharge, or signs of inflammation. A complete excisional biopsy with a surgical margin of 5 mm was performed under local anesthesia (Figure 1). Postoperative recovery was uncomplicated, and the surgical site showed good healing at two weeks (Figure 2). The specimen was submitted for histopathological analysis.

**Figure 3:**
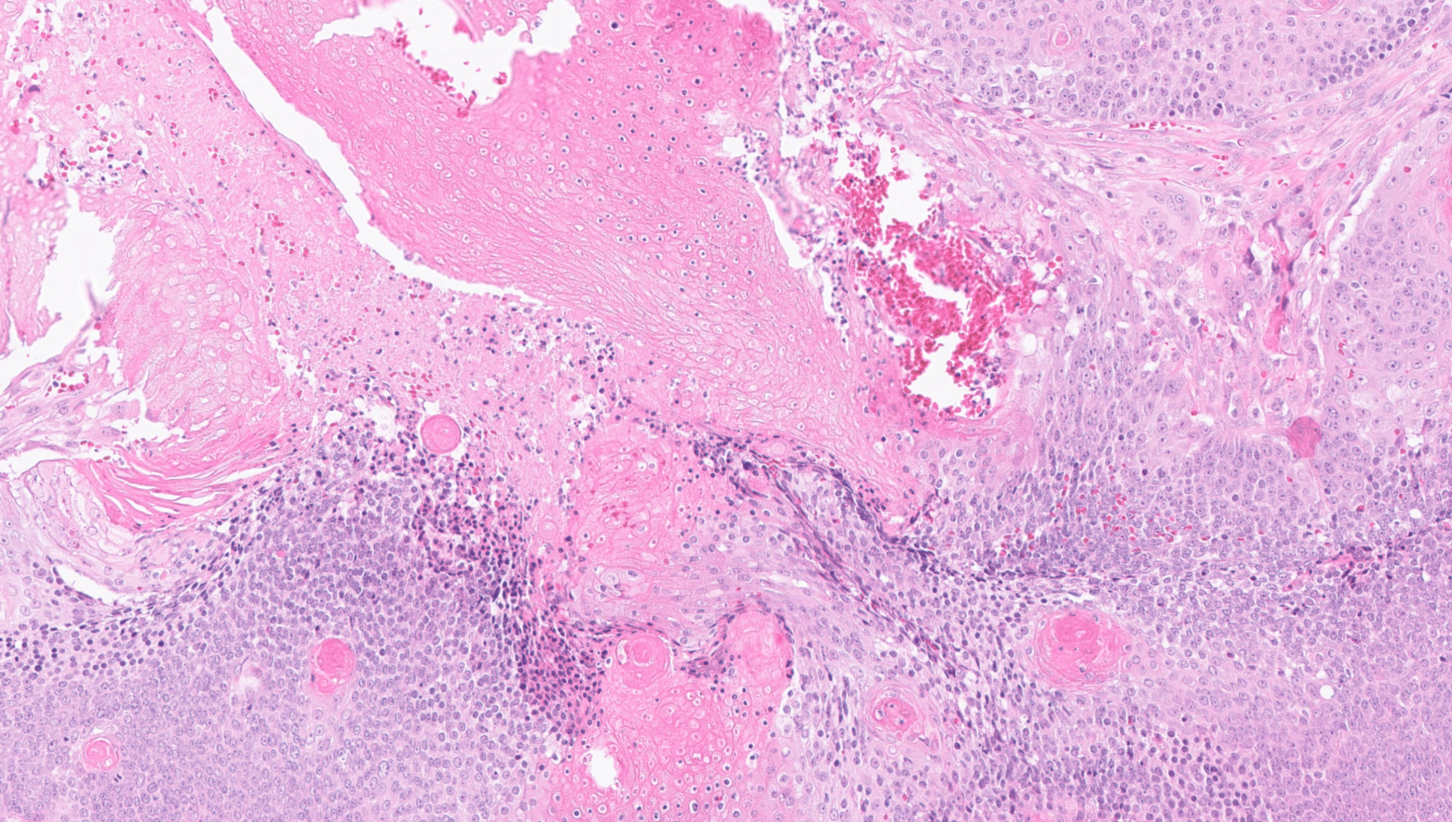
Histological section stained with Hematoxylin and Eosin (H&E) at ×200 magnification. The section shows characteristic basaloid cells and ghost cells.

Histopathological findings were consistent with pilomatrixoma, showing mummified squamous epithelium and peripheral basophilic cells without atypia or mitotic activity. The excised mass measured 0.8 x 0.4 x 0.3 cm, and surgical margins were histologically free of tumor (Figure 3).

The postoperative recovery was uncomplicated, and the patient showed no evidence of recurrence at one month.

## Discussion

Pilomatrixoma, though benign, can be misdiagnosed due to its rarity in older adults and its non-specific presentation. The literature reports a peak incidence in the first two decades of life, with a declining frequency in later years, and a higher prevalence in females [2-4]. However, recent reviews suggest a broader age distribution than previously believed. A systematic review analyzing excision ages reported a mean age of 16 years and 7 months, with a range spanning from 5 months to 97 years [3]. This highlights the importance of considering pilomatrixoma in the differential diagnosis of eyebrow masses, even in older patients, although occurrences after the age of 60 remain rare [3]. Maintaining an index of suspicion in this population remains essential, particularly when lesions appear in atypical locations or in patients with prior malignancies.

Although pilomatrixomas are benign lesions, rare cases of malignant transformation into pilomatrix carcinoma have been reported, most often following incomplete excision. Complete surgical removal with histopathological confirmation remains the standard treatment, given the benign nature of these tumors and the exceedingly low risk of malignancy [4]. Recurrence is uncommon - reported in fewer than 2% of cases - and typically results from incomplete resection [5,6].

Pilomatrix carcinoma is extremely rare, and due to its scarcity, there are no standardized treatment protocols [7]. Nonetheless, when it does occur, it is most often associated with prior incomplete excision. While routine follow-up is generally not necessary for benign pilomatrixomas, individual factors - such as a history of malignancy - may warrant closer clinical surveillance.

In elderly patients, a new or enlarging cutaneous nodule must prompt evaluation for malignancy, particularly in those with an oncological history. In our case, the history of breast carcinoma necessitated consideration of cutaneous metastasis. However, the lesion's features and histology were consistent with pilomatrixoma. This case contributes to the existing literature by highlighting a rare presentation of eyelid pilomatrixoma in an octogenarian patient with a history of breast carcinoma, raising important diagnostic considerations during oncological follow-up.

The differential diagnosis for lesions that mimic pilomatrixomas is extensive. It includes epidermal and dermoid cysts, atypical mycobacterial infections, parotid gland tumors, preauricular sinuses, ossifying hematomas, giant cell tumors, and chondromas. Other possibilities include foreign-body granulomas, calcified lymph nodes, and various benign or malignant soft tissue tumors [8-10].

Imaging such as ultrasound may aid in preoperative assessment, although it was not pursued in this case due to low clinical suspicion of malignancy. Nonetheless, high-resolution ultrasound could have been considered to assess internal characteristics such as calcifications or vascularity, particularly in elderly patients or when malignancy is a concern. CT, PET/CT, or MRI may also be appropriate when further lesion characterization is needed [3-8].

Given the patient's prior breast cancer, cutaneous metastasis remained an important differential diagnosis [11]. Although breast cancer is the most common malignancy in women, cutaneous metastases from this cancer remain relatively rare [12]. Nevertheless, they represent the most frequent cause of cutaneous metastases in this population. Therefore, any new or atypical skin lesion in a patient with a history of breast cancer should be carefully evaluated to rule out metastatic involvement. These metastases most commonly present as skin-colored nodules located on the anterior chest wall, contralateral breast, surgical scars, or arms - locations that are not typical of pilomatrixomas [13,14].

## Conclusions

The presentation of pilomatrixoma in elderly patients is exceptionally rare. Its diagnosis relies on clinical and histopathological examination, with surgical excision as the preferred treatment. In elderly patients with a history of cancer, thorough evaluation of any suspicious skin lesion is essential to exclude metastatic disease.
